# Noninvasive Portable Hemoglobin Concentration Monitoring System Using Optical Sensor for Anemia Disease

**DOI:** 10.3390/healthcare9060647

**Published:** 2021-05-29

**Authors:** Chuchart Pintavirooj, Baorong Ni, Chaiwat Chatkobkool, Kittitorn Pinijkij

**Affiliations:** 1 School of Engineering, King Mongkut Institute of Technology Ladkrabang, Bangkok 10520, Thailand; 59010113@kmitl.ac.th (C.C.); 59010311@kmitl.ac.th (K.P.); 2 Faculty of Engineering, Fukuoka Institute of Technology, Fukuoka 811-0295, Japan

**Keywords:** anemia, hemoglobin, Beer–Lambert, photoplethysmography

## Abstract

Anemia is a condition in which red blood cells are not able to carry adequate oxygen to the body’s tissues, and is widely found in nearly a quarter of the world population. The typical method to screen for the iron-deficiency anemia, which is the major anemia found in the world, is to implement a blood test called a complete blood count (CBC). However, even though this test gives a highly accurate result, it requires an invasive blood drawing and lab analyzing which could potentially cause physical pain, high risk of infection and take a long time to analyze. Therefore, this research presents an alternative method using an optical technique to measure hemoglobin concentration, which is the common indicator for diagnosing anemia. The light absorbance of the oxyhemoglobin at the wavelength of 660 nm and the deoxyhemoglobin at the wavelength of 880 nm were measured using the MAX30100 sensor. These wavelengths of light are obtained from red and infrared (IR) LEDs. The concept is based on the different absorption coefficients of blood at different electromagnetic wavelengths. This fact is used to indirectly calculate the hemoglobin concentration of blood through the modified Beer–Lambert law. Moreover, the result has been further converted to absolute hemoglobin concentration using a calibration curve derived from the cyanmethemoglobin test, which is the regular method for hemoglobin determination. Besides, the android application was also provided which can wirelessly record or monitor the data. The experiment shows that an accuracy of 90.9% can be achieved by our proposed noninvasive method. Therefore, the noninvasive portable hemoglobin concentration monitoring by the optical sensor has an acceptable result when compared with the invasive method, with less pain and lower risk of infection, as well as shorter processing time.

## 1. Introduction

Hemoglobin (Hb) is one of the most essential components of the human red blood cell. It is composed of four protein molecules, called globulin chains, and each globulin chain contains an important central structure called the heme molecule, which has iron embedded within [1,2]. Blood hemoglobin level is a vital clinical parameter for performing several blood tests. In cases where the Hb concentration falls below the defined level, this is called anemia [3]. Hemoglobin in red blood cells is responsible for carrying oxygen from the alveoli in the lung to all the cells and tissues within the body [1]. Thus, having anemia makes the body’s tissues fail to acquire a sufficient amount of oxygen, resulting in a variety of disorders such as tiredness, weakness, or even heart failure, which may lead to death [3]. Besides, according to the World Health Organization (WHO), around 24.8% of the world’s population suffers from anemia [4].

Traditionally, to diagnose anemia, a blood smear is performed to estimate complete blood counts (CBC), which can be done manually or automatically [5,6]. CBC is an invasive method to measure Hb concentration, which is a reliable indicator for anemia [7]. The disadvantage of blood smear is not only is it an uncomfortable method for the patient, but it is also a time-consuming process, taking a great portion of time between the blood collection and its analysis. Therefore, it does not allow real-time patient monitoring in crucial situations [6,8]. Near-infrared spectroscopy [9] has been proposed as a potential noninvasive method that could improve an unsatisfactory traditional method by allowing a pain-free continuous patient monitoring with minimum risk of infection [9,10]. Moreover, the method can be applied for real-time data monitoring. 

Near-infrared spectroscopy has been widely used for diagnosis and biomedical sensing. Due to the low-energy electromagnetic wave used, the technique is safe to be used for biological tissue. In general, spectroscopy requires the transmission of near-infrared light to the tissue, and it measures the transmitted light. The intensity of the transmitted light depends on the properties of the exposed tissue and blood hemoglobin concentration. To cancel the static properties of tissue, such as tissue thickness, normally, two wavelengths of light sources are used, which are in the visible and near-infrared range. The method is called the photoplethysmography method (PPG). PPG is the method used for measuring the transmission or reflection of light through the blood volume for observing the pulsatile changes of blood volume in the microvascular bed of the tissue [11,12]. Thus, blood plasma volume can be calculated, leaving only the absorption of the hemoglobin. Furthermore, Rajashree et al. [13] have reported the non-invasive optical method of hemoglobin determination by using NIR transmission at the wavelengths of 660 and 940 nm, which therefore shows that the capabilities of these selected wavelengths are promising for hemoglobin measurement. This optical sensor system uses wavelengths of light for the measurement of Hb concentration. This non-invasive optical measurement method is based on radiation of red and near-infrared light at the ranges of 660 and 940 nm, respectively [14,15]. The detector detects the transmitted light through the finger. To achieve the mathematical conversion from the detected light intensity at different wavelengths to hemoglobin concentration, the modified Beer–Lambert law is used and further improved by readjusting the outcome respective to the result from the cyanmethemoglobin method [16]. 

This paper presents a noninvasive hemoglobin concentration monitoring method using the optical sensor by studying the light absorbance of hemoglobin at the wavelengths of 660 and 880 nm, and subsequently using the modified Beer–Lambert law to calculate the relative hemoglobin concentration. To further improve outcomes, we create a calibration curve by readjusting the modified Beer–Lambert outcomes respective to the result from the hemoglobin cyanide method. The salient aspects and/or contributions of this paper are as follows:

(1) This paper presents the development of a novel, low-cost, portable, easy-to-use, fast and painless hemoglobin concentration monitoring device for anemia disease.
(2) This system is made noninvasive by using an optical sensor and requires no other set-up for analysis. As a result, the system is safe and flexible.
(3) The system provides both an LCD display and a Bluetooth connection. The analyzed results could be shown and stored on a smartphone via the Bluetooth connection.

This paper is organized as follows: Section 2 is devoted to materials and methods, covering the Beer–Lambert law, data acquisition and signal condition, the mathematical implementation and hemoglobin calculation software utilizing Beer–Lambert law, the calibration process with the cyanmethemoglobin method, statistic tools and the portable hemoglobin concentration monitoring system. Section 3 presents the experimental results. Lastly, the discussion and conclusion are presented in Section 4.

## 2. Materials and Methods

Our proposed noninvasive hemoglobin concentration measurement is based on the well-known Beer–Lambert law, where a light source is aimed onto a material. The attenuation of light intensity is related to the properties of the material in which the light is traversing through. The modified Beer–Lambert law has been used to describe light propagation through biological tissue and applied to measure tissue blood volume and oxygenation. Our proposed technique is based on the modified Beer–Lambert law, which is further applied for measuring the change in total hemoglobin concentration. 

### 2.1. Beer–Lambert Law

**Figure 1:**
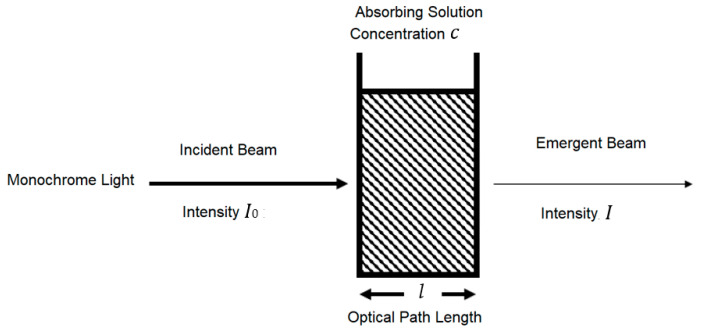
Relationship between the attenuation of light through a substance and the properties of that substance.

A standard and practical expression of the Beer–Lambert law relates the optical attenuation of a physical material containing a single attenuating species of uniform concentration to the optical path length through the sample and absorptivity of the species, as shown in Figure 1. The expression is *A* = *εcl* = *Log* (*I*_0_/*I*), where ε is the molar attenuation coefficient or absorptivity of the attenuating species, *l* is the optical path length, *c* is the concentration of the attenuating species, *I*_0_ is the light intensity of incident light and *I* is the light intensity of the received light. A general form of the Beer–Lambert law states that, for *n* attenuating species in the material sample, $A=\left[\sum_{i=1}^{n}\varepsilon_{i}\left(\lambda \right)C_{i}\right]$l [17,18].

#### 2.1.1. Beer–Lambert Law along Non-Linear Mean Light Pathways

**Figure 2:**
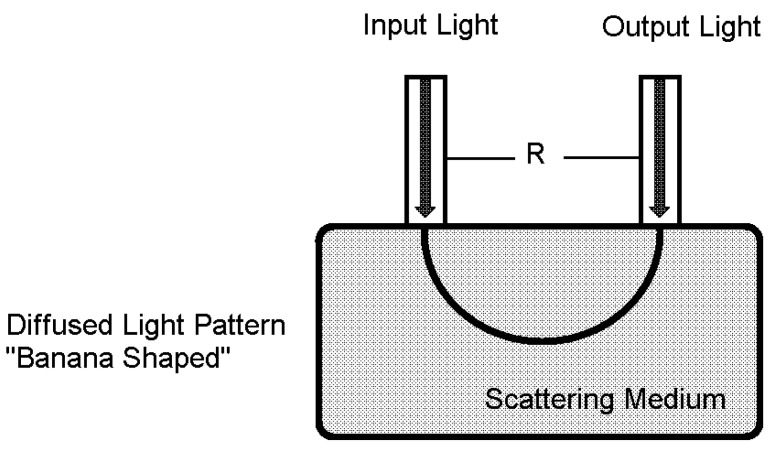
Banana-shaped envelope used in the Beer–Lambert law, where light detector and emitter are aligned on the same side.

The light flux envelope among detector and emitter inside a scattering substance such as vascular tissue, called “banana”, is shown in Figure 2. This banana-shaped envelope displays an empty space that most photons could be traveling along to the light detector from the light emitter [19]. The banana-shaped envelope is widely used for Beer–Lambert law application. 

#### 2.1.2. Modified Beer–Lambert Law (MBLL)

The common approach of continuous-wave near-infrared spectroscopy applied more than one light source–detector pair to the illuminated tissue as being optically homogeneous, which can be described by the modified Beer–Lambert law as:

*A* = *Log* (*I*_0_/*I*) =*εcl* + *G* (1)

where *G* is a geometry-dependent factor, which is independent of absorption and shows intensity loss caused by scattering. The differential form of the MBLL (dMBLL) still holds for relatively tiny changes of attenuation, if *G* can be regarded as constant, and the absorption changes homogeneously in the illuminated tissue volume:

(2) $$\Delta A=Log(\Delta I_{0}/\Delta I)=\varepsilon \Delta cl$$

where Δ*A* represents a shift in absorbance, Δ*I*_0_ and Δ*I* are the change in the light intensity of incident light and the light intensity of received light respectively, and Δ*c* is a change of the concentration of the attenuating species.

Due to multiple scattering, the physical pathlength traveled by the light through tissue is considerably higher than the source–detector separation employed in the measurement system. To consider this, a multiplicative term known as differential pathlength factor (*DPF*) was introduced to relate the actual light pathlength with the source–detector separation distance, *d*, as follows:

*l* = *d·DPF* (3)

(4) ΔA=Log(ΔI0/ΔI)=ε·Δc·d·DPF

The term *d* corresponds to the mean light propagation distance in the medium, and the parameter *DPF*(*λ*) is a scaling factor that indicates how many times farther than *d* the detected light has traveled.

Based on the diffusion equation for modeling light transport through a homogeneous semi-infinite medium, it can be shown that the *DPF* depends on the absorption coefficient *μ_a_*(*λ*), the reduced scattering coefficient *μ’_s_*(*λ*) and *d,* described by Equation (5):

(5) DPF(λ)=12(3μs′(λ)μa(λ))12[1−1(1+d(3μa(λ)μs′(λ))12)]≈12(3μs′(λ)μa(λ))12

Consequently, *DPF(λ)* increases with $\mu_{s}^{\prime }$ and decreases with $\mu_{a}$. The dependence of *DPF* on the source–detector separation (*d*) is crucial to be considered for small *d* values, but not for *d* > 2.5 cm. The *DPF* is virtually independent of *d*. From the mathematical point of view, when *d >* 2.5 cm, the dependence of *d* on the *DPF* is negligible, i.e., the inequality $d\sqrt{3\mu_{a}\mu_{s}^{\prime }}$ ≫ 1 holds, and hence the term 1(1+d(3μa(λ)μs′(λ))12) approaches zero. Since Equation (5) is only valid for a homogeneous semi-infinite medium, and since the human tissue is inhomogeneous, the equation only gives an approximation of the real situation in human tissue. However, the conclusion about the dependence of the DPF on *μ’_s_* and *μ_a_* remains true. For biological tissue, *DPF(λ)* is generally in the range of 3 to 6 [18].

Recently, Scholkmann researched the general equation for the differential pathlength factor of the frontal human head depending on wavelength and age, and showed *DPF* values at 660 and 880 nm for different wavelengths from many studies [19].

### 2.2. Data Acquisition and Signal Conditioning 

MAX30100 is a pulse oximetry [20,21] and heart rate module that is designed for wearable devices. It consists of an 880 nm infrared light emission diode (IR LED), 660 nm red light emission diode (Red LED) and photodetector and signal conditional circuit, including Direct Current (DC)-optimized optics, DC Ambient Light Rejection and low-noise analog signal processing [22]. MAX30100 is widely used in a minimum-power consumption wearable device that can provide a high-accuracy measurement of peripheral oxygen saturation (SpO2) and heart rate (HR). To optimize performance for various sites of measurement, LED current and LED pulse can be adjusted. The adjustable ranges of LED current and LED pulse are 0 to 50 mA and 200 μs to 1.6 ms, respectively. 

However, although MAX30100 provides all the essential components, the output coming from MAX30100 still needs to be accustomed. The output signal must be modified through the following libraries. 

The first library concerns DC removal. To remove the DC component, signal averaging is estimated at some instance of time and later subtracted from the original signal. DC removal can be carried out by applying the infinite impulse response filter (IIR). 

The second library is used to balance IR and Red currents and is used to control the intensity of IR and Red. A mechanism of this library is to first check the difference between *IDCred* and *IDCIR* readings. After that, if the value of *Ired* is greater than *I_IR_*, then it will decrease the *Ired* current, but if the *Ired* value is less than the *I_IR_*, then it will increase the *Ired* current. 

**Figure 3:**
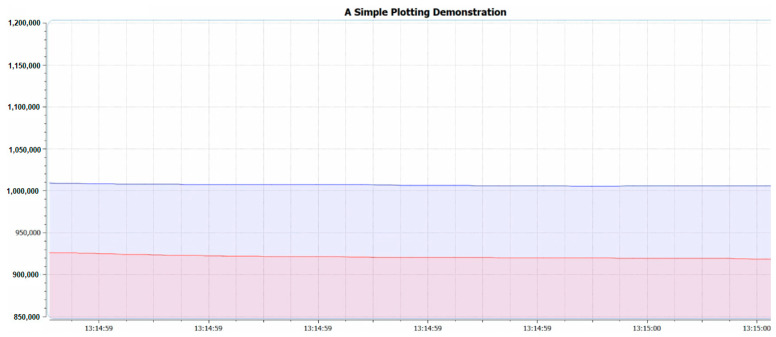
MAGIC_ACCEPTABLE_INTENSITY_DIFF (MAID) is too large.

**Figure 4:**
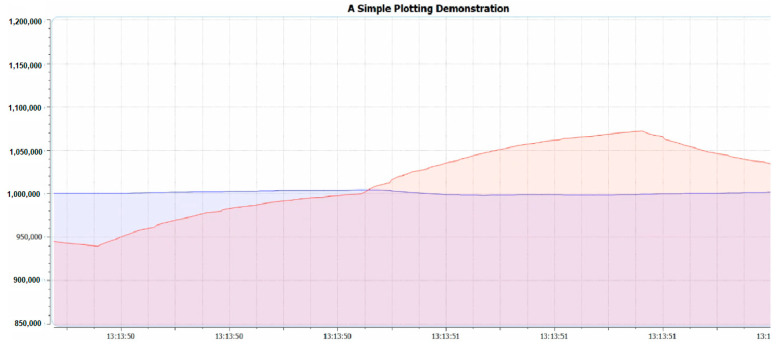
MAGIC_ACCEPTABLE_INTENSITY_DIFF is too small.

**Figure 5:**
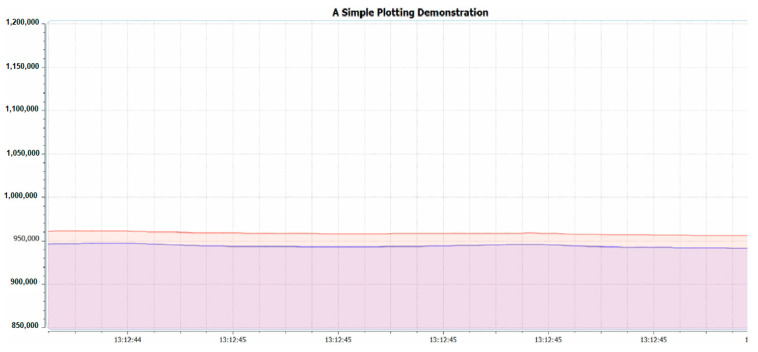
MAGIC_ACCEPTABLE_INTENSITY_DIFF is suitable.

The third library is the variable referring to the difference between the electrical current level supply of Red and IR light emitters or *IDCred* and *IDCIR,* which is called MAGIC_ACCEPTABLE_INTENSITY_DIFF (MAID). The suitable value of the MAID is important because if the MAID is too high, the oxygen saturation level will be oversaturated, as shown in Figure 3. However, on the other hand, if the MAID is too low, as shown in Figure 4, oscillation will occur. Lastly, when the MAID is suitable, the *IDCred* and *IDCIR* will balance out and stay stable, as shown in Figure 5.

The fourth library is low-pass filter (LPF), or the so-called mean filter. The aim of LPF is to smoothen the signal and hence improve the ability to detect the pulse of the signal. 

The last library is Butterworth band-pass filter (BPF), which is used to allow only the signal in the desired frequency to pass through, while removing the high-frequency signal, especially the higher-level harmonies of the signal. BPF also improves the performance of pulse detection by filtering the IR signal. 

After the signal goes through the aforementioned libraries, it is ready to be used by other libraries to find several biological values. 

Beat detection is the library that is used to determine human heart rate. To determine heart rate, signal thresholding is applied. When the signal reaches the setting threshold, the starting beat timestamp is marked. The signal is followed until the signal reaches the threshold again. The new timestamp is then marked as the ending beat timestamp. BPM is then calculated as: BPM = 60,000 ms/(ending beat timestamp − starting beat timestamp).

Ratio I library is used to estimate ratio *R* according to the principle of a pulse oximeter, which is defined as *R* = *log* (*IACred*)*·λ*1/*log*(*IACIR*)*·λ*2, where *IACred* is the root mean squared Red signal, *λ*1 is the Red wavelength at 660 nm, *IACIR* is the root mean squared IR signal and *λ*2 is the IR wavelength at 880 nm

For oxygen saturation (SpO2), a standard model for computing SpO2 is as follows: SpO2 = 110.0 − 25 × *R*. 

### 2.3. Mathematical Implementation and Hemoglobin Calculation Software

For the hemoglobin calculation software, the Beer–Lambert law is utilized and developed. The notation of absorbance to express light absorption as a function of hemoglobin concentration is given in Equation (6):

(6) $$A=Log\;\left(\frac{I_{0}}{I}\right)=\varepsilon cd\left(DPF\right)+G$$

where *A* is the absorbance, *I*_0_ is the light intensity of incident light, *I* is the light intensity of transmitted light, ε is the extinction coefficient of hemoglobin, *c* is the concentration of hemoglobin, *d* is the distance between the LED and optical sensor, *DPF* is the differential pathlength factor and *G* is a geometry of the measurement system [23,24]. However, *G* can be assigned as a constant, and if the absorption changes homogeneously in the illuminated tissue volume, the formula can be modified into:

(7) $$\Delta A=Log(\Delta I_{0}/\Delta I)=\varepsilon \cdot \Delta c\cdot d\cdot DPF$$

where Δ*A* represents a change in absorbance, Δ*I*_0_ and Δ*I* are the change in the light intensity of incident light and the light intensity of received light respectively, and Δ*c* is a change of the concentration of the attenuating species.

The MAX30100 sensor generally employs dual-wavelength LEDs to measure oxyhemoglobin and deoxyhemoglobin. Therefore, a general form of the modified Beer–Lambert law for a heterogenous material sample can be applied, as in Equation (8):

(8) $$\Delta A^{\lambda }=(\varepsilon_{HHb}^{\lambda }\Delta \left[HHb\right]DPF_{HbO2}^{\lambda }+\varepsilon_{HbO2}^{\lambda }\Delta \left[HbO2\right]DPF_{HbO2}^{\lambda })d^{\lambda }$$

where $\Delta A^{\lambda }$ represents a change in absorbance at wavelength λ nm, $\varepsilon_{HHb}^{\lambda }$ and $\varepsilon_{HbO2}^{\lambda }$ are the extinction coefficients of deoxyhemoglobin and oxyhemoglobin at wavelength λ nm respectively, Δ[HHb] and Δ[HbO2] are the change in deoxyhemoglobin concentration and oxyhemoglobin concentration at wavelength λ nm respectively, $d^{\lambda }$ is the distance between the λ nm LED and the optical sensor and $DPF_{HbO2}^{\lambda }$ is the differential pathlength factor of deoxyhemoglobin and oxyhemoglobin at wavelength λ nm.

Then, Equation (8) can be modified to be at wavelengths of 660 and 880 nm, as in Equations (9) and (10), where λ1 is 660 nm, λ2 is 880 nm and $\varepsilon_{HbO2}^{\lambda 1}$ is 693.44 cm^−1/M^, $\varepsilon_{HHb}^{\lambda 1}$ is 3226.56 cm^−1/M^ [25], $d^{\lambda 1}$ is 0.251 mm and $d^{\lambda 2}$ is 0.145 mm, as retrieved from the MAX30100 datasheet, and $DPF^{\lambda 1}$ is 7 and $DPF^{\lambda 2}$ is 8, as retrieved from Scholkmann’s general equation for the differential pathlength factor of the frontal human head depending on wavelength and age:

(9) $$\Delta A^{\lambda 1}=(\varepsilon_{HHb}^{\lambda 1}\Delta \left[HHb\right]DPF^{\lambda 1}+\varepsilon_{HbO2}^{\lambda 1}\Delta \left[HbO2\right]DPF^{\lambda 1})d^{\lambda 1}$$

(10) $$\Delta A^{\lambda 2}=(\varepsilon_{HHb}^{\lambda 2}\Delta \left[HHb\right]DPF^{\lambda 2}+\varepsilon_{HbO2}^{\lambda 2}\Delta \left[HbO2\right]DPF^{\lambda 2})d^{\lambda 2}$$

The Equation (9) can be rewritten into two forms:

(11) $$\Delta \left[HbO2\right]=\frac{\left(\frac{\Delta A^{\lambda 1}}{d^{\lambda 1}}\right)-\varepsilon_{HHb}^{\lambda 1}\Delta \left[HHb\right]DPF^{\lambda 1}}{\varepsilon_{HbO2}^{\lambda 1}DPF^{\lambda 1}}$$

(12) $$\Delta \left[HHb\right]=\frac{\left(\frac{\Delta A^{\lambda 1}}{d^{\lambda 1}}\right)-\varepsilon_{HbO2}^{\lambda 1}\Delta \left[HbO2\right]DPF^{\lambda 1}}{\varepsilon_{HHb}^{\lambda 1}DPF^{\lambda 1}}$$

The Δ[HbO2] from Equation (11) is replaced in Equation (10) and rewritten to be Equation (13):

$$\frac{\Delta A^{\lambda 2}}{d^{\lambda 2}}=\varepsilon_{HHb}^{\lambda 2}\Delta \left[HHb\right]DPF^{\lambda 2}+\frac{\varepsilon_{HbO2}^{\lambda 2}}{\varepsilon_{HbO2}^{\lambda 1}DPF^{\lambda 1}}\left(\frac{\Delta A^{\lambda 1}}{d^{\lambda 1}}-\varepsilon_{HHb}^{\lambda 1}\Delta \left[HHb\right]DPF^{\lambda 1}\right)DPF^{\lambda 2}$$

$$\frac{\Delta A^{\lambda 2}}{d^{\lambda 2}}=\varepsilon_{HHb}^{\lambda 2}\Delta \left[HHb\right]DPF^{\lambda 2}+\frac{\varepsilon_{HbO2}^{\lambda 2}\Delta A^{\lambda 1}DPF^{\lambda 2}}{\varepsilon_{HbO2}^{\lambda 1}DPF^{\lambda 1}d^{\lambda 1}}-\frac{\varepsilon_{HbO2}^{\lambda 2}\varepsilon_{HHb}^{\lambda 1}\Delta \left[HHb\right]DPF^{\lambda 1}DPF^{\lambda 2}}{\varepsilon_{HbO2}^{\lambda 1}DPF^{\lambda 1}}$$

$$\frac{\Delta A^{\lambda 2}}{d^{\lambda 2}}-\frac{\varepsilon_{HbO2}^{\lambda 2}\Delta A^{\lambda 1}DPF^{\lambda 2}}{\varepsilon_{HbO2}^{\lambda 1}DPF^{\lambda 1}d^{\lambda 1}}=\left(\varepsilon_{HHb}^{\lambda 2}DPF^{\lambda 2}-\frac{\varepsilon_{HbO2}^{\lambda 2}\varepsilon_{HHb}^{\lambda 1}DPF^{\lambda 1}DPF^{\lambda 2}}{\varepsilon_{HbO2}^{\lambda 1}DPF^{\lambda 1}}\right)\Delta \left[HHb\right]$$

$$\frac{\Delta A^{\lambda 2}\varepsilon_{HbO2}^{\lambda 1}DPF^{\lambda 1}d^{\lambda 1}-d^{\lambda 2}\varepsilon_{HbO2}^{\lambda 2}\Delta A^{\lambda 1}DPF^{\lambda 2}}{\varepsilon_{HbO2}^{\lambda 1}DPF^{\lambda 1}d^{\lambda 1}d^{\lambda 2}}=\left(\frac{\varepsilon_{HHb}^{\lambda 2}DPF^{\lambda 2}\varepsilon_{HbO2}^{\lambda 1}DPF^{\lambda 1}-\varepsilon_{HbO2}^{\lambda 2}\varepsilon_{HHb}^{\lambda 1}DPF^{\lambda 1}DPF^{\lambda 2}}{\varepsilon_{HbO2}^{\lambda 1}DPF^{\lambda 1}}\right)\Delta \left[HHb\right]$$

$$\left(\frac{\Delta A^{\lambda 2}\varepsilon_{HbO2}^{\lambda 1}DPF^{\lambda 1}d^{\lambda 1}-d^{\lambda 2}\varepsilon_{HbO2}^{\lambda 2}\Delta A^{\lambda 1}DPF^{\lambda 2}}{\varepsilon_{HbO2}^{\lambda 1}DPF^{\lambda 1}d^{\lambda 1}d^{\lambda 2}}\right)\cdot \left(\frac{\varepsilon_{HbO2}^{\lambda 1}DPF^{\lambda 1}}{\varepsilon_{HHb}^{\lambda 2}DPF^{\lambda 2}\varepsilon_{HbO2}^{\lambda 1}DPF^{\lambda 1}-\varepsilon_{HbO2}^{\lambda 2}\varepsilon_{HHb}^{\lambda 1}DPF^{\lambda 1}DPF^{\lambda 2}}\right)=\Delta \left[HHb\right]$$

(13) $$\Delta \left[HHb\right]=(\frac{\Delta A^{\lambda 2}\varepsilon_{HbO2}^{\lambda 1}DPF^{\lambda 1}d^{\lambda 1}-\varepsilon_{HbO2}^{\lambda 2}\Delta A^{\lambda 1}DPF^{\lambda 2}d^{\lambda 2}}{d^{\lambda 1}d^{\lambda 2}DPF^{\lambda 1}DPF^{\lambda 2}\left(\varepsilon_{HHb}^{\lambda 2}\varepsilon_{HbO2}^{\lambda 1}-\varepsilon_{HbO2}^{\lambda 2}\varepsilon_{HHb}^{\lambda 1}\right)}$$

Additionally, Δ[HHb] from Equation (12) is replaced in Equation (10) and rewritten to be Equation (14):

$$\frac{\Delta A^{\lambda 2}}{d^{\lambda 2}}=\varepsilon_{HbO2}^{\lambda 2}\Delta \left[HbO2\right]DPF^{\lambda 2}+\frac{\varepsilon_{HHb}^{\lambda 2}}{\varepsilon_{HHb}^{\lambda 1}DPF^{\lambda 1}}\left(\frac{\Delta A^{\lambda 1}}{d^{\lambda 1}}-\varepsilon_{HbO2}^{\lambda 1}\Delta \left[HbO2\right]DPF^{\lambda 1}\right)DPF^{\lambda 2}$$

$$\frac{\Delta A^{\lambda 2}}{d^{\lambda 2}}=\varepsilon_{HbO2}^{\lambda 2}\Delta \left[HbO2\right]DPF^{\lambda 2}+\frac{\varepsilon_{HHb}^{\lambda 2}\Delta A^{\lambda 1}DPF^{\lambda 2}}{\varepsilon_{HHb}^{\lambda 1}DPF^{\lambda 1}d^{\lambda 1}}-\frac{\varepsilon_{HHb}^{\lambda 2}\varepsilon_{HbO2}^{\lambda 1}\Delta \left[HbO2\right]DPF^{\lambda 1}DPF^{\lambda 2}}{\varepsilon_{HHb}^{\lambda 1}DPF^{\lambda 1}}$$

$$\frac{\Delta A^{\lambda 2}}{d^{\lambda 2}}-\frac{\varepsilon_{HHb}^{\lambda 2}\Delta A^{\lambda 1}DPF^{\lambda 2}}{\varepsilon_{HHb}^{\lambda 1}DPF^{\lambda 1}d^{\lambda 1}}=\left(\varepsilon_{HbO2}^{\lambda 2}DPF^{\lambda 2}-\frac{\varepsilon_{HHb}^{\lambda 2}\varepsilon_{HbO2}^{\lambda 1}DPF^{\lambda 1}DPF^{\lambda 2}}{\varepsilon_{HHb}^{\lambda 1}DPF^{\lambda 1}}\right)\Delta \left[HbO2\right]$$

$$\frac{\Delta A^{\lambda 2}\varepsilon_{HHb}^{\lambda 1}DPF^{\lambda 1}d^{\lambda 1}-d^{\lambda 2}\varepsilon_{HHb}^{\lambda 2}\Delta A^{\lambda 1}DPF^{\lambda 2}}{\varepsilon_{HHb}^{\lambda 1}DPF^{\lambda 1}d^{\lambda 1}d^{\lambda 2}}=\left(\frac{\varepsilon_{HbO2}^{\lambda 2}DPF^{\lambda 2}\varepsilon_{HHb}^{\lambda 1}DPF^{\lambda 1}-\varepsilon_{HHb}^{\lambda 2}\varepsilon_{HbO2}^{\lambda 1}DPF^{\lambda 1}DPF^{\lambda 2}}{\varepsilon_{HHb}^{\lambda 1}DPF^{\lambda 1}}\right)\Delta \left[HbO2\right]$$

$$\left(\frac{\Delta A^{\lambda 2}\varepsilon_{HHb}^{\lambda 1}DPF^{\lambda 1}d^{\lambda 1}-d^{\lambda 2}\varepsilon_{HHb}^{\lambda 2}\Delta A^{\lambda 1}DPF^{\lambda 2}}{\varepsilon_{HHb}^{\lambda 1}DPF^{\lambda 1}d^{\lambda 1}d^{\lambda 2}}\right)\cdot (\frac{\varepsilon_{HHb}^{\lambda 1}DPF^{\lambda 1}}{\varepsilon_{HbO2}^{\lambda 2}DPF^{\lambda 2}\varepsilon_{HHb}^{\lambda 1}DPF^{\lambda 1}-\varepsilon_{HHb}^{\lambda 2}\varepsilon_{HbO2}^{\lambda 1}DPF^{\lambda 1}DPF^{\lambda 2}})=\Delta \left[HbO2\right]$$

(14) $$\Delta \left[HbO2\right]=(\frac{\Delta A^{\lambda 2}\varepsilon_{HHb}^{\lambda 1}DPF^{\lambda 1}d^{\lambda 1}-\varepsilon_{HHb}^{\lambda 2}\Delta A^{\lambda 1}DPF^{\lambda 2}d^{\lambda 2}}{d^{\lambda 1}d^{\lambda 2}\left(\varepsilon_{HbO2}^{\lambda 2}DPF^{\lambda 2}\varepsilon_{HHb}^{\lambda 1}DPF^{\lambda 1}-\varepsilon_{HHb}^{\lambda 2}\varepsilon_{HbO2}^{\lambda 1}DPF^{\lambda 1}DPF^{\lambda 2}\right)}$$

The change of total hemoglobin concentration can be calculated by combining Δ[HHb] and Δ[HbO2] from Equations (13) and (14), as shown in Equation (15):

(15) Δ[Hb]=Δ[HHb]+Δ[HbO2]

After the modified Beer–Lambert law has been successfully adjusted, next, the raw signal must be modified as well. The MAX30100 sensor provides a DC digital filter to filter out the DC part of the signal. However, the AC part of the signal has still oscillated. Therefore, root mean square is used to find a mean of the signal and further subtract the data to lessen the pulse oscillation.

### 2.4. Calibration with Cyanmethemoglobin Method 

To validate our proposed method of hemoglobin concentration measurement using the modified Beer–Lambert law, we will compare the results with the standard method for laboratory determination of hemoglobin concentration in blood samples, namely the cyanmethemoglobin method [26]. 

#### 2.4.1. Cyanmethemoglobin Method 

Cyanmethemoglobin is the standard method for laboratory determination of hemoglobin concentration in blood samples. The test is normally performed by dissolving 20 microliters of blood in 5 mL of Drabkin’s solution. In addition, a spectrophotometer and calibration graph are used to estimate the hemoglobin concentration.

**Figure 6:**
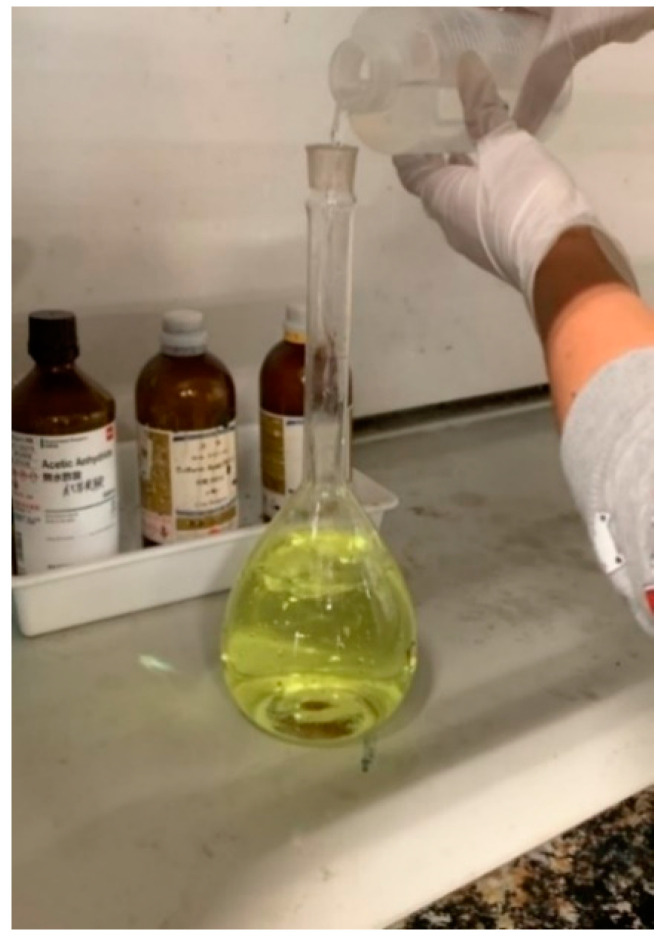
Preparation of a liter of Drabkin’s solution in a volumetric flask.

For this experiment, several solutions must be prepared, such as Drabkin’s solution, cyanmethemoglobin standard solution and diluted cyanmethemoglobin standard solution. We first prepared the Drabkin’s solution by mixing 1 vial of Drabkin’s reagent with 1 L of distilled water. Then, we added 0.5 mL of the Brij L23 solution. For safety reasons, due to the acidic properties of the solution, this process must be performed in the fume hood, as shown in Figure 6.

**Figure 7:**
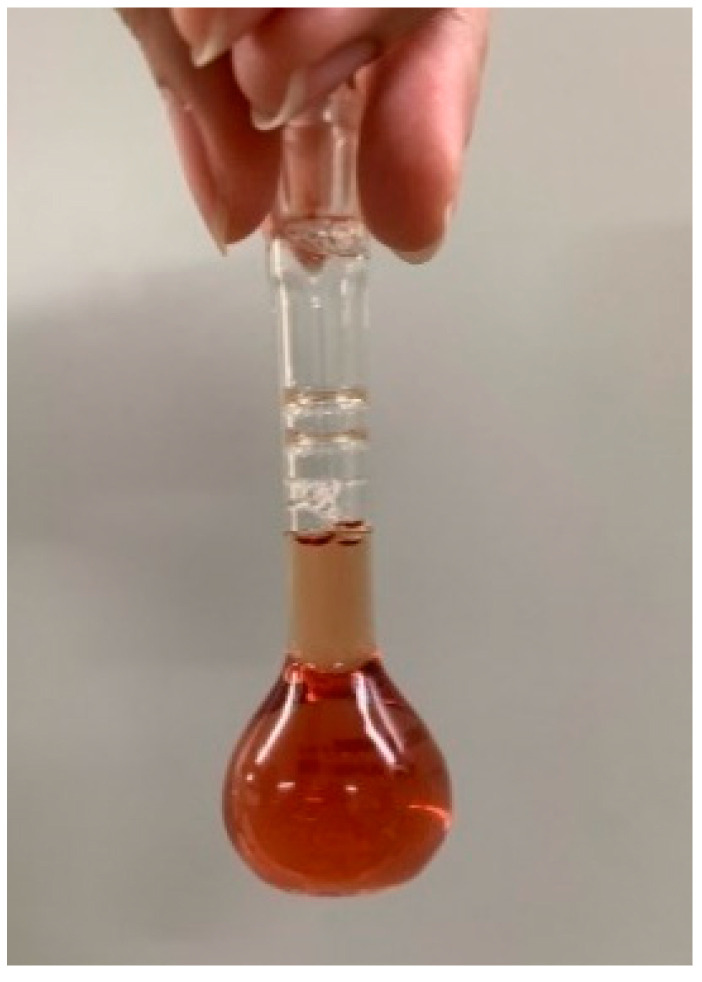
20 mL of cyanmethemoglobin standard solution in a volumetric flask.

The next step is to prepare the cyanmethemoglobin standard solution. This solution can be prepared by mixing 20 mL of Drabkin’s solution with 14.345 mg of hemoglobin human lyophilized powder, as shown in Figure 7.

Lastly, for the diluted cyanmethemoglobin standard solution, the solution can be created by mixing Drabkin’s solution with cyanmethemoglobin standard solution at different concentrations, as shown in Table 1. The decreased concentration of hemoglobin is needed to provide a calibration curve for hemoglobin concentration. 

After all the diluted cyanmethemoglobin standard solutions have been prepared, next, spectrophotometry must be performed. The spectrophotometer that has been used is a Hitachi u-2910 model, run with the software called UV solutions. In the software, the range of wavelength and type of data must be selected. The wavelength that was chosen between 450 and 650 nm as the interesting wavelength was 540 nm, and the type of data is absorbance. Besides, before measuring the data, the baseline must be adjusted first. Then, after all the samples were measured by the spectrophotometer, to validate the results, the samples were used to measure hemoglobin concentration with our proposed modified Beer–Lambert-based technique using the MAX30100 sensor. This can be carried out by transferring the solution to a small tube and measuring its absorbance in the darkroom.

However, the sensor, the MAX30100 pulse oximeter, is a reflectance sensor, so the received signal from the sensor is the reflectance of the light. Thus, the data must be converted before being used. Therefore, the relationship between absorbance of light and reflectance of light can be expressed as:

(16) $$Light\;Absorbance=Log\left(\frac{Total\;light\;intensity}{Light\;Reflectance\;}\right)$$

Nonetheless, this system contains a light-balancing intensity program and there are 2 different types of light, so the total light intensities are also varied by the amounts of currents that were used. For a 16-bit analog-to-digital converter and the maximum 50 mA current used, the total intensity of light will be equivalent to 65,535. For the current less than 50 mA, the total intensity of red and infrared light can be described by the following equations, respectively:

(17) $$Total\;Intensity\;of\;red\;light=\left((Current_{Red}-50)\;\cdot \left(\frac{65,535}{50}\right)\right)+65,535$$

(18) $$Total\;Intensity\;of\;infrared\;light=\left((Current_{Inf}-50)\;\cdot \left(\frac{65,535}{50}\right)\right)+65,535$$

where the convention factor is 65,535 per 50 mA. 

The light absorbance of the MAX30100 sensor can be calculated from Formulae (16) and (17). However, all the data were collected when the current of the red light was equal to 50 mA. By replacing $Current_{Red}$ in Equation (17) to 50 mA, total light intensity will be 65,535. Therefore, Equation (16) can be modified to:

(19) $$Light\;Absorbance=Log\left(\frac{65,535}{Light\;Reflectance\;}\right)$$

All measured data have been tabulated in Table 2, including hemoglobin concentration, MAX30100 sensor shifted light absorbance and light absorbance spectrophotometer. Note that the MAX30100 sensor shifted light absorbance is the shift of the MAX30100 sensor light absorbance such that the minimum is at 0. 

The calibration graph equation is further calculated by using minimized mean square error (MMSE) to estimate the linear equation between spectrophotometer light absorbance and hemoglobin concentration, as shown in Equation (20):

*Y* = 458.33*x* + 1.14436 (20)

where *x* is the spectrophotometer light absorbance and *y* is hemoglobin concentration.

#### 2.4.2. Statistical Analysis

After all the data were collected, 3 statistics tools were used to test the reliability of the data, which are F-test, *T*-test and linear regression. This process was carried out by using the MATLAB application.

##### F-Test Statistic

For this test, the result from the shifted MAX30100 absorbance value will be compared to the spectrophotometer absorbance value to test whether the result is coming from a single population or not. The F-test result for *N* = 3 is shown in Table 3.

The result has shown that all data are coming from a single population since all F values are less than the value from the F-table (at *n* = 1 and *d* = 4), which is 7.71. Thus, the data can be used interchangeably (*p* < 0.05).

##### Linear Regression and *T*-test Statistic

**Figure 8:**
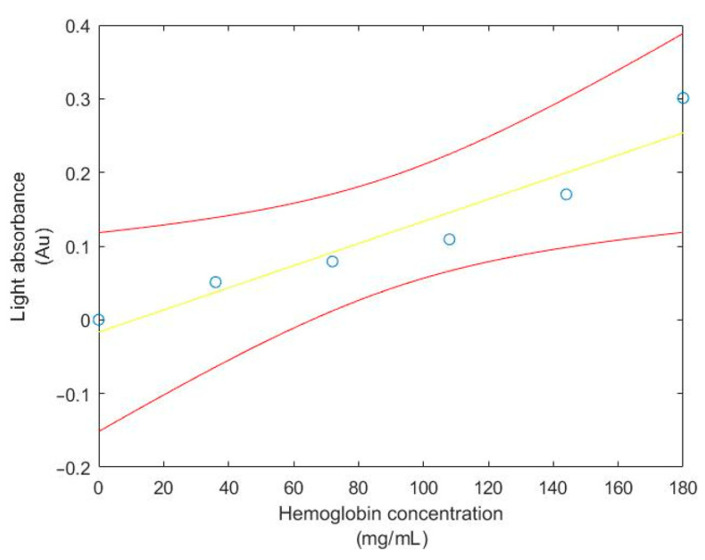
The 95% confidence interval of the regression line relating Hb concentration and light absorbance from the MAX30100 sensor.

**Figure 9:**
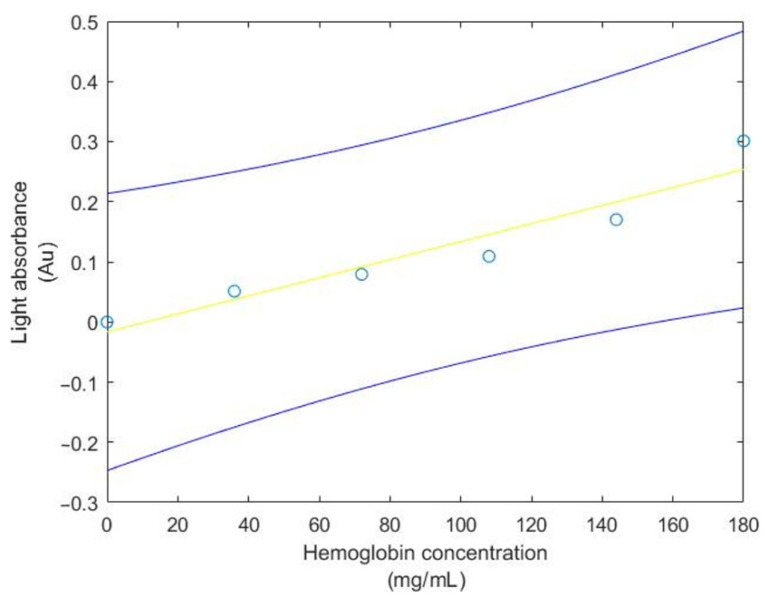
The 95% confidence interval for an additional observation of light absorbance from the MAX30100 sensor at given Hb concentration.

For this test, the shifted MAX30100 absorbance value and hemoglobin concentration will be used to find the regression line, the 95% confidence interval of the regression line, observation, correlation coefficient and coefficient of determination. Moreover, the *T*-test will be used to test whether the hemoglobin concentration and light absorbance from the MAX30100 sensor are corelated. The result of the 95% confidence interval of the regression line and observation between Hb concentration and light absorbance from the MAX30100 sensor are shown in Figure 8 and Figure 9, respectively. 

Results of the T-Test and coefficient of determination from MATLAB are given as:
Hemoglobin con(X)/Light ab(Y)SigX = 540.0000SigY = 0.710900SigX^2^ = 71,280.000000SigXY = 98.046000a = −0.016695b = 0.001502N^2^ = 6 Stdx = 67.349833Stdy = 0.106036Syx = 0.076230Sa = 0.044171Sb = 0.0005060.000097 < beta < 0.002907−0.169851 < alpha < 0.136460R = 0.954000r^2^ = 0.910117*t*-test = 6.364127Reject 6.364127 > 2.776000

From the *T*-test results, it can be seen that the hemoglobin concentration and light absorbance from the MAX30100 sensor are not coming from the same population, since the value of *T* = 6.1969, which is greater than the value from the T-table (at *v* = 4), which is 2.776. Thus, the hemoglobin concentration and light absorbance from the MAX30100 sensor are correlated (*p* < 0.05). Moreover, the coefficient of determination (*r^2^*) is equal to 0.910117, which means that around 91% of data can be explained by the graph.

#### 2.4.3. Calibration Software

Calibration software will be used by our system to determine the hemoglobin concentration. The hemoglobin concentration will be determined indirectly by creating a calibration curve. To obtain the calibrated data, 9 blood samples have been drawn from the three subjects at different times. We then measured hemoglobin concentration using a calibration graph derived from the cyanmethemoglobin method, i.e., light absorbance is measured using a spectrophotometer and later converted to hemoglobin concentration, as shown in Table 4. At the same time, modified Beer–Lambert data using our system were also collected for the 9 blood samples. Minimized mean square error (MMSE) was then used to fit the liner regression line between hemoglobin concentration and modified Beer–Lambert data. The derived regression line equation is provided in Equation (21), with *r^2^* of 0.924431, which demonstrates the linear relation between hemoglobin concentration and modified Beer–Lambert data:

*Calibrated data Value* = (*Modified Beer*–*Lambert Value* × 0.007) + 84.0701 (21)

Using the regression line from Equation (21), we will generate calibration data that will be used in our system. By varying the hemoglobin concentration from 0 to 180 mg/mL and recomputing the corresponding modified Beer–Lambert data, the re-sampling of hemoglobin concentration will be called the calibrated data value. Using the data in Table 2, the shifted MAX30100 absorbance value and spectrophotometer reflectance value are also interpolated. All calibration data are provided in Table 5.

Similar to data derived from the cyanmethemoglobin method, we also tested the reliability of the data using the F-test, *T*-test and linear regression. 

**Figure 10:**
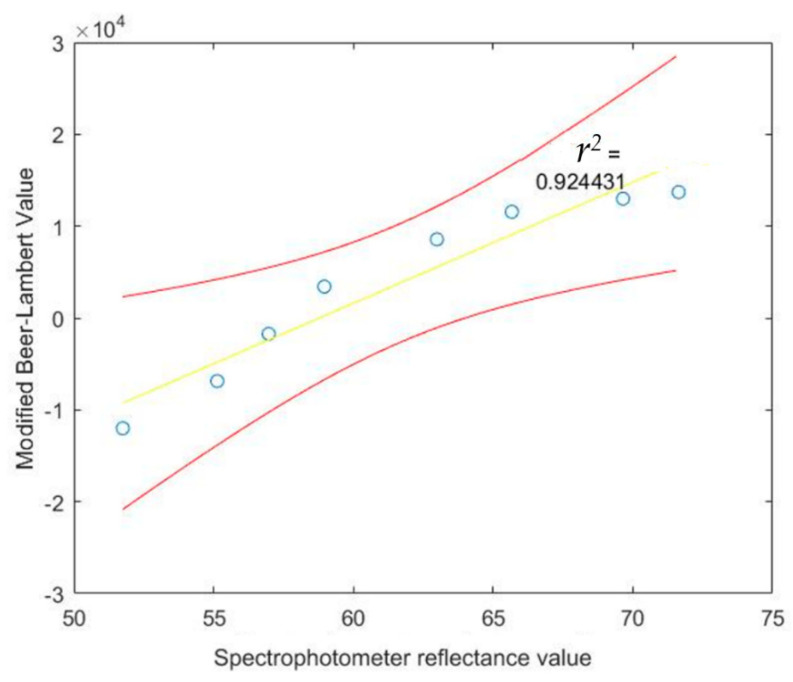
The 95% confidence interval of the regression line relating spectrophotometer reflectance value at difference Hb concentrations and calibrated value.

**Figure 11:**
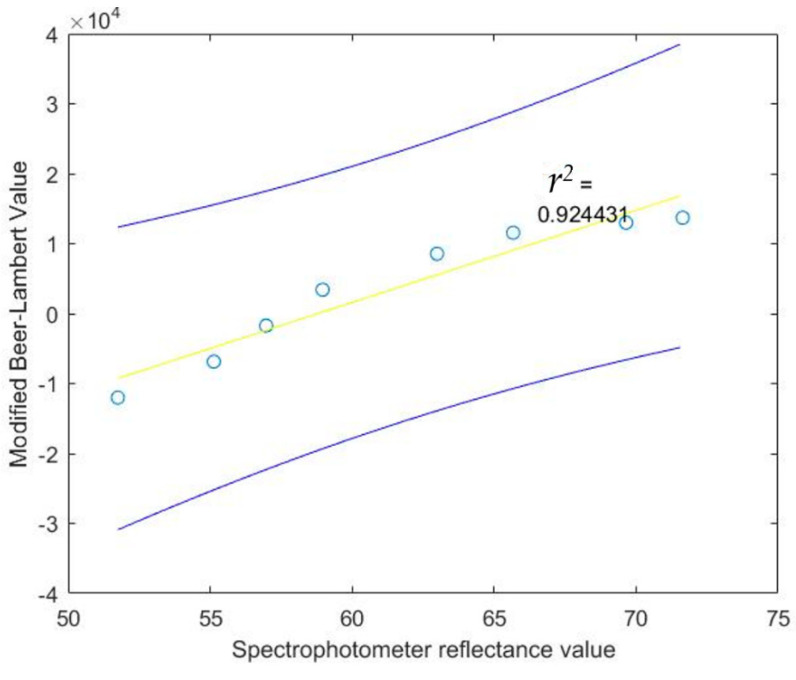
The 95% confidence interval for an additional observation of calibrated value at given spectrophotometer reflectance values.

The calibrated data value will be used to find the regression line, the 95% confidence interval of the regression line, observation, correlation coefficient and coefficient of determination. Moreover, we used a *T*-test to test whether the hemoglobin concentration and calibrated data value sensor are correlated. The results of the 95% confidence interval of the regression line and observation between Hb concentration and calibrated data are shown in Figure 10 and Figure 11, respectively.

Results of the *T*-test and coefficient of determination from MATLAB comparing the calibration graph and calibrated values are given as follows:

Spectrophotometer Reflectance Value(X)/Modified Beer–Lambert law Value (Y)
SigX = 492.760000SigY = 29,633.000000SigX^2^ = 30,702.943000SigXY = 2,288,190.110000a = −77,445.200858b = 1317.466123
N^2^ = 8Stdx = 7.085103Stdy = 9708.416900Syx = 7469.542827Sa = 24,685.608552Sb = 398.472935342.402852 < beta < 2292.529394−137,850.884985 < alpha < −17,039.516731
R = 0.961473r^2^ = 0.924431*t*-test = 8.567232Reject 8.567232 > 2.447000

From the *T*-test result, we can conclude that both hemoglobin concentration and calibrated data are not coming from the same population, since the value of *T* = 8.5672, which is greater than the value from the T-table (at *v* = 6), which was 2.447. Thus, the hemoglobin concentration and light absorbance from the MAX30100 sensor correlate (*p* < 0.05). Moreover, the coefficient of determination (*r^2^*) is equal to 0.924431, which means that around 92% of data can be explained by the graph.

After the calibration graph is finished, the calibrating software is developed. The software will provide the status of the tester based on the hemoglobin concentration level, with an error of 15%. The status is as shown below in Table 6.

### 2.5. Portable Hemoglobin Concentration Monitoring System

**Figure 12:**
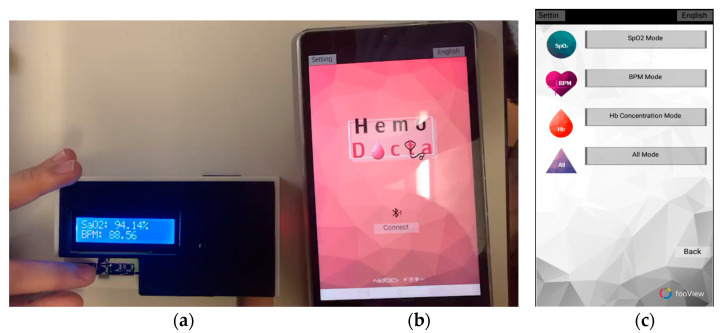
Portable hemoglobin concentration monitoring system: (**a**) hardware, (**b**,**c**) software.

A portable hemoglobin concentration monitoring system is shown in Figure 12, consisting of hardware and software. As shown in Figure 12a, hardware mainly consists of an ESP32 microcontroller, LCD display and a MAX30100 sensor. The hardware will be enclosed in a black plastic box to prevent surrounding light that could interfere with the red and near-infrared light sources. To provide wireless control of our portable hemoglobin concentration monitoring system, an android application using the MIT App invertor was written. The communication between the hardware and software parts uses a Bluetooth connection. Figure 12b shows the first page of the application, and Figure 12c shows the operating page, which consists of three modes: Spo2 mode, BPM mode and Hb concentration mode. To operate, choose the preferred mode and then simply press the index finger on the sensor for a while until the parameter is shown on the device or smart phone. In Hb concentration mode, the MAX30100 sensor will detect the light reflectance, convert to light absorbance and digitally filter out the DC signal. The AC signal of light absorbance was further calculated via the modified Beer–Lambert’s law and displayed the hemoglobin concentration on the LCD and smartphone.

## 3. Results

We conducted experiments with 11 volunteers. All volunteer subjects were Thai and in the age range of 22–26 years old, and the cohort consisted of 9 men and 2 women. All volunteers had no health problems, except one woman who was diagnosed with moderate anemia symptoms.

The subjects were restricted from having alcohol and caffeine for 12–24 h before the test and did not perform any physical activity or stress prior to the tests. Moreover, subjects were suggested to calm down and relax for 10–15 min and think about nothing during the test period.

In the beginning, the subject placed a finger on the sensor on the device so that the sensor can read the data and show it on the display. The data were collected for a least 30 s, 3 times for each person per mode. All the data are displayed in Table 7, Table 8 and Table 9. Moreover, the subject information is displayed in Table 10.

The expected result in the SpO2 table is the normal saturated oxygen level.

The expected result in the heartbeat table was measured from counting the pulse on the subject’s wrist.

The expected result in the hemoglobin concentration table was calculated according to various factors, consisting of their sex, age, race and congenital disorders [26].

## 4. Discussion 

Anemia is a disease concerning a decrease in hemoglobin concentration due to a reduction in hemoglobin synthesis. Anemia can be caused by several factors, including nutritional deficiencies and infectious diseases. In nutritional deficiency-related anemia, insufficient intake of iron, vitamin A, vitamin B12, pyridoxine and folate have been reported to contribute. Elderly people might easily suffer from anemia because of a decline in protein-calorie nutrition. In infectious disease-related anemia, patient with diseases interfering with plasma volume could result in a reduction in the circulating concentration of hemoglobin, as the hemoglobin concentration is defined as the quantity of hemoglobin in one unit of plasma volume. Patients with chronic heart failure, renal failure, liver failure and excessive sodium may suffer from an increase in circulating volume and hence a decrease in hemoglobin concentration. Patients with inflammatory intestine disease may suffer from enteric blood loss and finally, result in anemia. In normal people, hemoglobin concentration may be varied due to many reasons, including age, diastolic pressure, pulse pressure, albumin level and pharmacotherapy. Due to the variation of hemoglobin concentration, the WHO has classified the level of anemia based on the range of hemoglobin concentration. In normal males, hemoglobin concentration is 140–180 g/L, whereas in normal females, hemoglobin concentration is between 120 and 160 g/L. The standard clinically approved method for hemoglobin concentration measurement is called the cyanmethemoglobin method, in which a blood sample is drawn from a subject. Our proposed method of hemoglobin concentration measurement using the modified Beer–Lambert law is not intended to replace the cyanmethemoglobin method, rather, it is intended to be used as a screening test for anemia. Patients with negative results or at the boundary line will be encouraged to do further tests with a conventional cyanmethemoglobin hemoglobin concentration measurement method at the hospital. Moreover, as the proposed method is noninvasive and painless, it can be used for real-time monitoring of hemoglobin concentration.

## 5. Conclusions

In this paper, we proposed a noninvasive hemoglobin concentration monitoring by the optical sensor for anemia disease diagnosis, which improved the disadvantages of the invasive traditional hemoglobin concentration measuring method by allowing a pain-free continuous patient monitoring with minimum risk of infection, and reducing the amount of toxic waste in the process. Based on the MAX30100 pulse oximeter sensors to detect signals that pass through the human finger, we applied the modified Beer–Lambert law, which is derived to estimate the relative hemoglobin concentration level. To convert the absolute hemoglobin concentration, we created the calibration curve between the modified Beer–Lambert output and the standard measurement of hemoglobin concentration, known as the cyanmethemoglobin method. A large amount of in vitro data of hemoglobin absorbance at a concentration between 0 to 180 g/L was collected by using a spectrophotometer. The device failed to obtain the correct value of the SaO2, however, it obtained a precise value of the heartbeat with an error of 8%, and provided an acceptable result of hemoglobin concentration with an accuracy of 90.9%, within 15% of the acceptable error range.

## Figures and Tables

**Table 1:** Diluted cyanmethemoglobin standard solution at different concentrations.

Tube No.	Hemoglobin Concentration (mg/mL)	Drabkin’s Solution (mL)	Cyanmethemoglobin Standard Solution (mL)
1	0	5	0
2	36	4	1
3	72	3	2
4	108	2	3
5	144	1	4
6	180	0	5

**Table 2:** Light absorbance values from all devices.

Hemoglobin Concentration	MAX30100 Sensor Light Reflectance	MAX30100 Sensor Light Absorbance	MAX30100 Sensor Light Absorbance (Shifted)	Light Absorbance Spectrophotometer
0	10,800	0.7830	0	00
36	9600	0.8342	0.0512	0.0740
72	9000	0.8622	0.0792	0.1517
108	8400	0.8922	0.1092	0.2313
144	7300	0.9532	0.1702	0.3103
180	5400	1.0841	0.3011	0.3920

**Table 3:** F-test result from MATLAB for *N* = 3.

Hemoglobin Concentration	F-Test	F-Table
36	7.05	7.71
72	7.38	7.71
108	6.74	7.71
144	7.13	7.71
180	5.63	7.71

**Table 4:** Data measured by the modified Beer–Lambert value and cyanmethemoglobin method.

Cyanmethemoglobin Method	Modified Beer–Lambert Value
176	4308
173.4	4745
175.6	4443
159.6	9358
161.5	8920
162.2	8716
107.9	14,111
109.1	13,344
107	14,540

**Table 5:** Calibrated data.

MAX30100 Absorbance Value	Spectrophotometer Reflectance Value	Modified Beer–Lambert Value	Calibrated Data Value
−0.0025	51.75	−12,010	0
0.0760	55.13	−6867	36
0.1546	56.97	−1724	72
0.2331	58.96	3418	108
0.0303	62.99	7990	140
0.3117	65.67	8561	144
0.3467	69.65	10,847	160
0.3900	71.64	13,704	180

**Table 6:** The anemia diagnosis status *.

Severity	Hemoglobin Concentration (g/L)
Normal	120–160 for women 140–180 for men
Mild	100 to normal limit
Moderate	80–100
Severe	65–79
Life-Threatening	<65

* Adapted from [27].

**Table 7:** SpO2 measured data and expected result.

SpO2					
	Measured Data (%)			Average (%)	Expected Result (%)
	Round 1	Round 2	Round 3		
Subject 1	94.44	94.14	94.40	94.33	>95
Subject 2	94.03	94.63	94.45	94.37	>95
Subject 3	93.64	93.54	93.58	93.59	>95
Subject 4	94.26	94.66	93.98	94.29	>95
Subject 5	94.54	93.60	94.24	94.13	>95
Subject 6	94.51	94.50	94.21	94.41	>95
Subject 7	94.59	93.87	94.66	94.37	>95
Subject 8	94.50	94.51	94.91	94.64	>95
Subject 9	93.96	93.87	93.86	93.90	>95
Subject 10	94.90	94.60	95.02	94.84	>95
Subject 11	89.31	85.40	86.12	86.94	>95

**Table 8:** Heartbeat measured data and expected result.

Heartbeat					
	Measured Data (BPM)			Average (BPM)	Expected Result (BPM)
	Round 1	Round 2	Round 3		
Subject 1	82 (6.82%)	92 (4.55%)	88 (0.00%)	87 (1.14%)	88
Subject 2	65 (99%)	66 (1.49%)	70 (4.47%)	67 (0.00%)	67
Subject 3	84 (1.18%)	86 (1.18%)	88 (3.53%)	86 (1.18%)	85
Subject 4	88 (0.00%)	90 (27%)	85 (3.41%)	88 (0.00%)	88
Subject 5	72 (1.41%)	72 (1.41%)	75 (5.63%)	73 (82%)	71
Subject 6	98 (4.56%)	90 (4.56%)	93 (1.06%)	94 (0.00%)	94
Subject 7	85 (0.00%)	88 (3.53%)	85 (0.00%)	87 (35%)	85
Subject 8	79 (3.66%)	82(0.00%)	83 (1.22%)	81 (1.22%)	82
Subject 9	90 (3.22%)	92 (1.01%)	96 (3.22%)	93 (0.00%)	93
Subject 10	85 (0.00%)	80 (5.88%)	91 (7.06%)	85 (0.00%)	85
Subject 11	96 (13%)	94 (0.00%)	94 (0.00%)	95 (1.06%)	94

**Table 9:** Hemoglobin concentration measured data and expected result.

Hemoglobin Concentration							
	Measured Data (g/L)			Average (g/L)	Actual Blood Test (g/L)	Expected Result (g/L)	Status
	Round 1	Round 2	Round 3				
Subject 1	200.48 (14.56%)	169.55 (3.21%)	153.25 (12.43%)	174.43 (0.33%)	175.00	140–180	Normal
Subject 2	168.03 (3.09%)	144.98 (11.05%)	151.89 (6.82%)	152.79 (6.26%)	163.00	140–180	Normal
Subject 3	164.63 (1.42%)	171.28 (56%)	179.98 (7.77%)	171.96 (97%)	167.00	140–180	Normal
Subject 4	149.32 (41%)	168.78 (10.31%)	146.33 (4.36%)	154.81 (1.18%)	153.00	140–180	Normal
Subject 5	165.27 (1.63%)	169.61 (0.95%)	163.86 (46%)	166.25 (1.04%)	168.00	140–180	Normal
Subject 6	130.52 (16.87%)	133.09 (15.23%)	136.12 (13.30%)	133.24 (15.13%)	157.00	140–180	Normal
Subject 7	166.26 (0.16%)	167.16 (0.70%)	167.38 (0.83%)	166.92 (0.55%)	166.00	140–180	Normal
Subject 8	144.19 (10.44%)	149.52 (7.13%)	140.74 (12.58%)	144.82 (10.05%)	161.00	140–180	Normal
Subject 9	140.74 (10.36%)	132.38 (15.68%)	131.54 (16.22%)	134.89 (14.08%)	157.00	140–180	Normal
Subject 10	143.55 (4.93%)	141.40 (6.36%)	147.24 (55%)	144.06 (4.60%)	151.00	120–160	Normal
Subject 11	115.77 (7.49%)	113.18 (5.78%)	120.07 (12.21%)	116.34 (8.73%)	107.00	<110	Mild

**Table 10:** Subject information.

Subject Information				
	Sex	Age	Race	Hemoglobin-Related Disorder
Subject 1	Male	22	Asian (Thai)	-
Subject 2	Male	22	Asian (Thai)	-
Subject 3	Male	26	Asian (Thai)	-
Subject 4	Male	26	Asian (Thai)	-
Subject 5	Male	24	Asian (Thai)	-
Subject 6	Male	26	Asian (Thai)	-
Subject 7	Male	24	Asian (Thai)	-
Subject 8	Male	25	Asian (Thai)	-
Subject 9	Male	25	Asian (Thai)	-
Subject 10	Female	26	Asian (Thai)	-
Subject 11	Female	25	Asian (Thai)	Mild Anemia

## Data Availability

Data sharing not applicable.
